# Acute Neurological Manifestations in COVID‐19 Are Associated With Complement Activation and Glia‐Promoted Neuroinflammation

**DOI:** 10.1002/jmv.71165

**Published:** 2026-09-28

**Authors:** Nicole Lardini Freitas, Yago Côrtes Pinheiro Gomes, Fábio César Sousa Nogueira, Juliana Echevarria‐Lima, João Victor Carvalho Deus, Carlos Otávio Brandão, Cristiane Nascimento Soares, Marcus Tulius Teixeira Silva, Otávio Melo Espíndola

**Affiliations:** ^1^ Instituto Nacional de Infectologia Evandro Chagas Fundação Oswaldo Cruz Rio de Janeiro Brazil; ^2^ Instituto Oswaldo Cruz Fundação Oswaldo Cruz Rio de Janeiro Brazil; ^3^ Laboratório de Proteômica Laboratório de Apoio ao Desenvolvimento Tecnológico (LADETEC), Instituto de Química, Universidade Federal do Rio de Janeiro Rio de Janeiro Brazil; ^4^ Unidade de Proteômica Instituto de Química, Universidade Federal do Rio de Janeiro Rio de Janeiro Brazil; ^5^ Laboratório de Imunologia Básica e Aplicada Instituto de Microbiologia Paulo de Góes, Universidade Federal do Rio de Janeiro Rio de Janeiro Brazil; ^6^ Laboratório Neurolife Rio de Janeiro Brazil; ^7^ Departamento de Doenças Infecto Parasitárias Hospital Federal dos Servidores do Estado Rio de Janeiro Brazil; ^8^ Serviço de Neurologia Complexo Hospitalar de Niterói Niterói Brazil

## Abstract

Pathophysiological mechanisms of neurological manifestations in COVID‐19 are not fully known yet. In this case‐control study, proteomic analysis was used to characterize cerebrospinal fluid (CSF) samples from COVID‐19 patients with acute neurological manifestations primarily categorized as: isolated refractory headache (*n* = 12), encephalopathy (*n* = 24), and inflammatory neurological diseases (IND) (*n* = 13). Individuals with non‐inflammatory, non‐infectious neurological conditions (*n* = 8) were included as controls. Differentially expressed proteins (DEPs) in neuro‐COVID‐19 were associated with the complement system, function/activation of microglia/macrophages, inflammatory responses, and neuronal function/homeostasis. Functional enrichment analysis of DEPs and/or proteins specifically detected in neuro‐COVID‐19 groups indicated activation of complement and coagulation cascades, and synapse pruning in encephalopathy and IND. Upregulated complement activation was confirmed by increased CSF levels of C3a and soluble C5b‐9. Biological processes of neuroinflammation, migration of immune cells into the central nervous system, activation of glia cells (microglia and astrocytes), and tissue remodeling were also present in IND. In turn, processes associated with disturbed organization/formation of neuron projections were related to encephalopathy and isolated headache. CSF from patients with isolated refractory headache was enriched for proteins related to neurons and to the cerebral cortex, cerebellum, and basal ganglia. Meanwhile, CSF from encephalopathy and IND patients was mostly enriched for proteins mapped to the cerebral cortex, choroid plexus, and thalamus. Moreover, CSF from IND patients had an increased proportion of microglia‐related proteins. These findings support a model in which cases of IND and encephalopathy are associated with blood−brain barrier disruption potentially induced by complement activation and glia‐promoted neuroinflammation, resulting in the involvement of distinct brain structures in COVID‐19.

## 1 Introduction

Neurological manifestations have been frequently associated with COVID‐19 regardless of respiratory involvement. These neurological changes result from affected central and peripheral nervous systems, leading to a broad range of symptoms/signs, ranging from anosmia/ageusia and headache to acute complications, including cerebrovascular diseases, encephalopathy, coma, Guillain−Barré syndrome, and a plethora of inflammatory neurological diseases (IND) such as encephalitis, meningitis, meningoencephalitis, acute disseminated encephalomyelitis (ADEM), and transverse myelitis [1, 2, 3, 4, 5, 6, 7, 8, 9, 10, 11]. However, underlying mechanisms associated with the pathophysiology of neuro‐COVID‐19 are still unclear. Viral direct damage, autoimmunity, and indirect effects from systemic inflammation have been proposed and investigated as possible mechanisms of COVID‐19 neuropathogenesis [12, 13].

Neuroinvasion by SARS‐CoV‐2 has been shown by detection of viral RNA and proteins in the cerebrospinal fluid (CSF) and brain parenchyma [14, 15, 16]. However, central nervous system (CNS) infection seems limited as indicated by infrequent detection of the viral RNA in CSF samples [17] and the low viral load in the brain compared to other organ tissues, such as lungs, kidneys, and intestine [18]. Angiotensin‐converting enzyme 2 (ACE2), the cellular receptor for SARS‐CoV‐2, is expressed by neurons, astrocytes, and oligodendrocytes, particularly within the substantia nigra, ventricles, middle temporal gyrus, posterior cingulate cortex, and olfactory bulb, albeit at lower levels compared to the lungs [19]. Response to autoantigens has also been shown in cases of acute neuro‑COVID. Intrathecal production of anti‑SARS‑CoV‑2 IgG and CSF‑restricted oligoclonal bands (OCB) is observed in a small proportion of patients with encephalitis or encephalopathy [20, 21, 22]. In contrast, the investigation of CNS‑targeted autoantigens has revealed heterogeneous profiles, including autoantibodies against NMDAR, CASPR2, GD1a, LGI1, MYL7, UCH‐L1, GRIN3B, and other neuronal antigens [15, 23, 24, 25, 26, 27].

CSF and blood changes in patients with acute neuro‐COVID‐19 are diverse. CNS inflammatory syndromes in COVID‐19 can appear early (0−14 days from symptom onset), as a parainfectious process without significant systemic involvement, or even without confirmation of SARS‐CoV‐2 neuroinvasion [9, 17]. Moreover, encephalopathy cases, defined as altered mental status with normal CSF cell counts and without brain imaging signs of neuroinflammation, seem mostly influenced by systemic inflammation [28]. The analysis of altered CSF proinflammatory and regulatory cytokines, growth factors, and factors involved in cellular stress response in neuro‐COVID‐19 has shown common mechanisms in cases of encephalopathy and IND. These were primarily related to CNS cytokine storm, neuroinflammation, and microglial functions, leukocyte activation, proliferation, and chemotaxis, inflammasome activity, and possible disturbances of the blood−brain barrier (BBB), supporting a model in which both systemic and CNS inflammation contribute to the diversity of neurological outcomes [29].

In this study, CSF samples from patients with COVID‐19 previously characterized with distinct neurological outcomes, including isolated refractory headache, encephalopathy, and IND, were assessed by proteomic analysis to identify mechanisms likely involved in triggering CNS inflammation and acute neurological disturbances in COVID‐19. Functional enrichment analysis of proteins with altered expression in CSF showed that biological processes related to complement activation, blood coagulation disturbances, neuroinflammation with migration of immune cells and activation of resident CNS cells (microglia and astrocytes), tissue remodeling, and disturbed organization and formation of neuron projections were the main pathways involved in the presentation of distinct neurological outcomes in COVID‐19.

## 2 Materials and Methods

### 2.1 Study Population, Biological Samples, and Case Definitions

This case‐control study was conducted in accordance with STROBE recommendations [30]. The study was carried out with COVID‐19 patients displaying acute neurological manifestations admitted between April and June 2020 to reference hospitals in the cities of Rio de Janeiro (Hospital Federal dos Servidores do Estado and Instituto Nacional de Infectologia Evandro Chagas) and Niterói (Complexo Hospitalar de Niterói), Brazil. Sixty patients with SARS‐CoV‐2 infection confirmed by detection of viral RNA in nasopharyngeal swabs by reverse transcription quantitative PCR (RT‐qPCR) were eligible. Six patients presenting neurological alterations affecting the peripheral nervous system, such as Guillain−Barré and Miller−Fisher syndromes, and evidence of acute cerebrovascular disease in brain magnetic resonance imaging (MRI) or tomography, were excluded from the study. Two patients with a diagnosis of concurrent infection by other neuropathogens, as previously described [9], were also excluded. CSF samples obtained were immediately processed, aliquoted, and stored at −80°C until use. Lumbar punctures were atraumatic, and CSF samples had adequate red blood cell (RBC) counts (4 RBC/mm^3^, interquartile range = 0−33 RBC/mm^3^), except for two patients with encephalopathy who had 2048 and 5547 RBC/mm^3^. Three patients were excluded from this study due to insufficient CSF volume to perform assays, and paired serum samples were available for 31 of 49 participants included in the study.

As controls, CSF samples (*n* = 8) from unmatched patients with non‐inflammatory non‐infectious neurological diseases (normal pressure hydrocephalus) who underwent CSF withdrawal before the emergence of COVID‐19 in Brazil were included as a reference non‐infected (NI) group. These individuals had normal values for intracranial opening pressure (≤ 200 mmH_2_O), CSF cell counts (< 5 cells/mm^3^), and total proteins (< 45 mg/dL) [28, 29].

#### 2.1.1 Case Definitions

COVID‐19 patients were grouped according to their primary neurological condition, as previously characterized [6, 9, 28, 29]: (i) isolated refractory headache (*n* = 12), (ii) encephalopathy (*n* = 24), and (iii) IND (*n* = 13). Isolated refractory headache developed after a median interval of 2 days (IQR = 0–10 days), encephalopathy after 3 days (IQR = 2–7 days), and IND after 4 days (IQR = 0–14 days) following the onset of common COVID‐19 symptoms.

Isolated refractory headache was defined as a daily intense headache, occasionally accompanied by visual symptoms, and representing the sole reported central neurological complaint. Study participants who presented any clinical or laboratory evidence for meningitis or meningoencephalitis, such as neck stiffness, altered consciousness, focal neurological signs, or inflammatory characteristics in brain MRI and/or in the CSF analysis, were excluded from this group and allocated to the group of IND.

Encephalopathy was characterized by diffuse brain dysfunction, convulsions, alterations in cognition, consciousness, personality, or behavior, and without signs of acute CNS inflammation. Seizures and abnormalities on electroencephalogram (EEG) were considered for the diagnosis of both encephalopathy and encephalitis. However, to avoid overlapping between these groups, encephalopathy was defined as a syndrome of altered mental status (major criterion) without signs of acute brain inflammation, such as CSF pleocytosis and changes in brain MRI. To define encephalitis, in addition to altered mental status, it was required at least two of the following minor criteria: fever, new‐onset seizures not attributable to a preexisting seizure disorder, focal neurologic signs, CSF pleocytosis, acute abnormalities on neuroimaging, or EEG abnormalities indicative of cerebral inflammation, such as generalized or focal slow‐wave activity, electrographic seizures [31].

Other inflammatory neurological syndromes, including ADEM (*n* = 2), encephalitis (*n* = 3), meningitis (*n* = 2), meningoencephalitis (*n* = 2), acute myelitis (*n* = 3), and neuromyelitis optica (*n* = 1), were classified accordingly by experienced neurologists.

### 2.2 IgG Anti‐SARS‐CoV‐2 Spike Protein ELISA

CSF and serum samples were screened for IgG anti‐SARS‐CoV‐2 Spike protein (ancestral lineage) using an in‐house ELISA, as previously described [32]. The cut‐off was determined as the mean optical density (OD) of negative controls plus the standard deviation. Samples with OD/cut‐off ratio > 2.0 were considered positive, values between 1.0 and 2.0 were indeterminate, and samples with results < 1.0 were negative.

### 2.3 Proteomic Analysis

#### 2.3.1 Sample Preparation and Protein Digestion

At the time of use, CSF samples were thawed, and a volume corresponding to 100 μg of total proteins was concentrated in a SpeedVac centrifuge (Thermo Fisher Scientific, MA, USA) at room temperature. To enhance the sensitivity of the proteomic analysis, CSF samples were processed with the High Select Top 14 Abundant Protein Depletion Spin Columns (Thermo Fisher Scientific, MA, USA), following the manufacturer's instructions, to reduce the amounts of albumin (ALB), immunoglobulins (IgG, IgA, IgM, IgD, IgE), kappa and lambda light chains, alpha‐1‐acid glycoprotein (A1AG1), alpha‐1‐antitrypsin (SERPINA1), alpha‐2‐macroglobulin (A2M), apolipoprotein A1 (APOA1), fibrinogen (FGB), haptoglobin (HP), and transferrin (TF). Samples depleted of abundant proteins were again concentrated in a SpeedVac centrifuge and resuspended in 20 μL of 400 mM ammonium bicarbonate and 8 M urea. After, 5 μL of 100 mM dithiothreitol (DTT) was added, and the solution was incubated at 37°C for 1 h. Following, 5 μL of 400 mM iodoacetamide was added, and the solution was further incubated at room temperature and protected from light for 1 h. Finally, ammonium bicarbonate and urea concentrations were adjusted to 50 mM and 1 M, respectively, by adding 130 μL of deionized water (Milli‐Q), and protein digestion was performed overnight at 37°C with 1/50 (m/m) of sequencing grade modified trypsin (Promega, WI, USA).

#### 2.3.2 Liquid Chromatography‐Tandem Mass Spectrometry Analysis

Peptide solutions were desalted using Pierce Peptide Desalting Spin Columns (Thermo Fisher Scientific, MA, USA) according to the manufacturer's instructions. Peptide samples were dried in a SpeedVac centrifuge without heating and resuspended in 0.1% formic acid before separation using an EASY1000 Nano‐liquid chromatography system (Thermo Fisher Scientific, MA, USA). The flow rate was set to 300 nL/min with a 160 min linear gradient of phase B (95% acetonitrile and 0.1% formic acid). The gradient started at 5%−30% B for 112 min, followed by 10 min at 30%−38% B, 10 min at 38%−58% B, 5 min at 58%−93% B, and 23 min at 93% B. A 50 cm EASY‐SPRAY column, heated to 60°C, was used for separation.

Peptides were analyzed using a Q‐Exactive Plus Mass Spectrometer (Thermo Fisher Scientific, MA, USA). For the Ion Source, the spray voltage was set to 1900 V, and the ion transfer tube temperature was maintained at 250°C. For the master scan (MS1), it was used a resolution of 70 000, with a m/z scan range of 375−1650, and an AGC target of 3.0 × 10^6^, and an injection time of 100 ms. The 20 most intense ions were fragmented using high‐energy collisional dissociation (HCD) and a 200−2000 m/z range. The Orbitrap resolution was set to 17 500, AGC target of 1.0 × 10^6^, an isolation window of 1.4 m/z, and a normalized collision energy (NCE) of 30. Additionally, data‐dependent acquisition mode was performed to obtain MS2 spectra, with an intensity threshold set at 1.0 × 10^5^, an injection time of 50 ms, and a dynamic exclusion period of 40 s.

#### 2.3.3 Proteomic Data Analysis

Database search and peptide/protein identification were conducted using Fragpipe v.18.0. Searches were performed against a reviewed and concatenated *Homo sapiens* reference proteome downloaded from The UniProt Consortium database (2022), along with a list of contaminants provided by the software. The MSFragger algorithm was used with a precursor mass tolerance of 10 ppm and a fragment mass tolerance of 0.1 Da. Fixed and variable modifications followed the default settings of the LFQ‐MBR workflow. Full tryptic cleavage specificity was applied, allowing up to two missed cleavage sites. Protein quantification was based on MS1 precursor ion intensity using the IonQuant tool. The MaxLFQ method was selected, requiring at least one ion for protein quantification. The MBR tool was applied in default mode, with a false discovery rate (FDR) controlled at < 1%. FDR was calculated using Percolator, and only high‐confidence proteins were considered.

Proteins displaying intensity > 0 were considered as identified. For the quantitative analysis, the intensity values scale and normalization were carried out with the Perseus software version 1.6.10.43. For this, proteins identified in at least one sample of each group were used. The intensity values were log2‐transformed, and the missing values were input with values from a normal distribution and then normalized by subtracting each value from the median protein intensity among samples. The resulting data matrix was exported and further analyzed in the *R* software v4.4.2.

The workflow of CSF proteomic analysis is shown in the Supporting Information S1: Table S1 and Figure S1. Proteins detected in at least one sample or in one‐third (33%) of the samples of each study group were visualized with the VennDiagram package for R software and considered for further analysis where indicated. Principal component analysis (PCA) of proteomic profiles was performed with FactoMineR and factoextra packages, and differential expression analysis of CSF proteins was conducted with limma and ggrepel packages.

### 2.4 Quantification of Subproducts of Complement Activation

Activation of the complement system in the CNS was assessed by quantification of C3a and C5b‐9 in CSF samples. C3a was quantified by ELISA (BD OptEIA Human C3a ELISA Kit, BD Biosciences) with CSF samples diluted at 1:50, and soluble C5b‐9 was quantified by Luminex (MILLIPLEX Human Complement C5b‐9 Singleplex, Merck Millipore) with undiluted samples, following the manufacturer's instructions.

### 2.5 Data Analysis

Statistical analysis and design of graphs were conducted with R software v4.4.2 (https://cran.r-project.org/). Inflammatory neurological syndromes such as ADEM, encephalitis, meningitis, meningoencephalitis, acute myelitis, and neuromyelitis optica were combined into a single IND group to enhance statistical power for further analysis, despite their differences in clinical presentation. Data distribution was evaluated by the Shapiro−Wilk test. Comparative analysis of non‐parametric data was carried out with Kruskal−Wallis and post hoc analysis with Dunn's test with the Holm−Sidak method for adjusting *p* values in multiple comparisons. Data with normal distribution was evaluated by ANOVA followed by post hoc analysis with Tukey's test for three or more groups, or by Student's *t‐*test for two groups. Fisher's exact test was employed to evaluate differences in the distribution of qualitative data across groups. The annotation of biological functions was performed with terms retrieved from the databases of Gene Ontology (GO‐terms) and the Kyoto Encyclopedia of Gene and Genomes (KEGG) (hsa identifiers). Functional enrichment analysis was performed with the gprofiler2 package, and biological processes with the highest protein ratio were selected, prioritizing child terms over generalist parent terms, and displayed in dot plot graphs generated by the enrichplot package. CSF proteins identified within each study group, and those differentially expressed between neuro‐COVID‐19 patients and uninfected controls, were mapped to 13 CNS regions and 5 resident cell types using the Human Brain Atlas database. The analysis followed previously described approaches [33, 34] and used RNA expression data reported as normalized transcripts per million (nTPM), derived from RNA sequencing of micro‐dissected areas and regions of the human brain (https://www.proteinatlas.org/humanproteome/brain). The association with cell types and brain region was inferred by identifying proteins with elevated gene expression in a given CNS region or cell type. Specifically, a protein was assigned to a region or cell type when its corresponding gene showed expression levels at least fourfold higher than the mean nTPM across the remaining brain regions or cell types in the dataset. Proteins that did not meet this enrichment criteria in any CNS region or cell type were excluded from further analysis. Rose plots were generated based on the total number of mapped proteins according to neuro‐COVID‐19 groups using the ggplot2 package. Heatmaps were produced with the nTPM data in specific brain regions and CNS cell types retrieved from the Human Brain Atlas database according to neuro‐COVID‐19 groups using the pheatmap package.

## 3 Results

### 3.1 Characteristics of the Study Population

COVID‐19 patients were grouped according to their primary neurological condition as isolated refractory headache, encephalopathy, and IND [6, 9, 28, 29]. Most patients presented neurological manifestations within 14 days of COVID‐19 onset. Only three patients developed neurological alterations after this period: one with encephalopathy, one with ADEM, and one with acute myelitis, starting at Days 18, 19, and 29, respectively. CSF samples were collected at the time of clinical presentation of neurological symptoms, minimizing variability related to sampling time. Given the rarity of CSF samples from patients with acute neurological manifestations of COVID‐19, these cases were retained and explicitly indicated in subsequent analyses. Due to the limited sample size, no adjustment for time from COVID‐19 onset to neurological manifestation was performed.

Mean age differed across groups (ANOVA, *p* = 0.003); patients with isolated refractory headache were younger than those with encephalopathy and controls (Tukey's test, *p* = 0.004 and *p* = 0.010, respectively). Sex distribution also varied across groups (Fisher's exact test, *p* = 0.006), with a predominance of females among patients with isolated refractory headache and IND, and of males among those with encephalopathy. Nevertheless, neuro‐COVID‐19 patients overall did not differ from controls in age (*t*‐test, *p* = 0.220) or sex (Fisher's exact test, *p* = 0.700), further highlighting the heterogeneity across neurological presentations (Table 1).

**Table 1:** Characteristics of the study population.

Characteristics	Isolated refractory headache	Encephalopathy	IND	Total neuro‐COVID‐19	NI (control)	*p* value^e^
	(*n* = 12)	(*n* = 24)	(*n* = 13)	(*n* = 49)	(*n* = 8)	
Age (years)^a^	42.7 ± 9.6	61.0 ± 17.6	47.2 ± 14.2	52.9 ± 17.0	62.6 ± 19.6	**0.003**
Sex^b^	***n* (%)**	***n* (%)**	***n* (%)**	***n* (%)**	***n* (%)**	
Male	2 (16.7)	15 (62.5)	1 (7.7)	18 (36.7)	4 (50.0)	**0.006**
Female	10 (83.3)	9 (37.5)	12 (92.3)	31 (63.3)	4 (50.0)	
Symptoms^c^	***n* (%)**	***n* (%)**	***n* (%)**	***n* (%)**	***n* (%)**	
Fever	9 (75.0)	11 (45.8)	4 (30.8)	24 (49.0)	0 (0.0)	—
Headache	12 (100)	4 (16.7)	5 (38.5)	21 (42.9)	0 (0.0)	—
Cough	6 (50.0)	9 (37.5)	2 (15.4)	17 (34.7)	0 (0.0)	—
Anosmia/ageusia	5 (41.7)	8 (33.3)	4 (30.8)	17 (34.7)	0 (0.0)	—
Myalgia	8 (66.7)	2 (8.3)	2 (15.4)	12 (24.5)	0 (0.0)	—
Altered level of consciousness	1 (8.3)	9 (37.5)	2 (15.4)	12 (24.5)	0 (0.0)	—
Respiratory symptoms	4 (33.3)	6 (25.0)	1 (7.7)	11 (22.4)	0 (0.0)	—
Fatigue	3 (25.0)	3 (12.5)	2 (15.4)	8 (16.3)	0 (0.0)	—
Seizure	—	3 (12.5)	1 (7.7)	4 (8.2)	0 (0.0)	—
Paresthesia	—	—	3 (23.1)	3 (6.1)	0 (0.0)	—
Diarrhea	1 (8.3)	1 (4.2)	1 (7.7)	3 (6.1)	0 (0.0)	—
Paresis	—	—	2 (15.4)	2 (4.1)	0 (0.0)	—
Aphasia	—	1 (4.2)	—	1 (2.0)	0 (0.0)	—
COVID‐19 severity^d^						
Mild	12 (100)	9 (37.5)	6 (46.2)	27 (55.1)	n.a.	—
Moderate	0 (0.0)	2 (8.3)	1 (7.7)	3 (6.1)	n.a.	—
Severe	0 (0.0)	2 (8.3)	1 (7.7)	3 (6.1)	n.a.	—
Death	0 (0.0)	2 (8.3)	0 (0.0)	2 (4.1)	n.a.	—
Undefined	0 (0.0)	9 (37.5)	5 (38.5)	14 (28.6)	n.a.	—

Abbreviations: IND, inflammatory neurological diseases; n.a., not applicable; NI, non‐infected.

^a^ Values are presented as mean ± standard deviation. Statistical analysis was performed using ANOVA followed by post hoc analysis with Tukey's test.

^b^ Differences in sex distribution among groups were assessed using Fisher's exact test.

^c^ Detailed COVID‐19 symptoms in medical reports were available for 35 participants, including 12 with isolated refractory headache, 15 with encephalopathy, and 8 with IND. Altered level of consciousness included confusion, delirium, and somnolence. Respiratory symptoms included nasal congestion, dyspnea, and pulmonary involvement greater than 50%.

^d^ COVID‐19 severity was defined according to the WHO guidelines [34].

^e^ Differences with a *p* < 0.05 were considered statistically significant (in bold).

Most patients presented mild COVID‐19 respiratory symptoms (55.1%) and were primarily hospitalized for the investigation of neurological manifestations (Table 1). Fever and headache were the most frequent symptoms, reported in 49.0% and 42.9% of patients, respectively, followed by cough and anosmia/ageusia (34.7%). One‐fourth of the patients presented with myalgia and altered level of consciousness (24.5%), which included confusion, delirium, and somnolence, followed by respiratory symptoms (22.4%), including nasal congestion, dyspnea, and pulmonary involvement with ground‐glass opacities exceeding 50% on chest computed tomography, and fatigue (16.3%). Less frequent symptoms included seizures (8.2%), paresthesia and diarrhea (6.1%), in addition to paresis (4.1%) and aphasia (2.0%) (Table 1).

### 3.2 Presence of IgG Anti‐Spike in Serum but Not in CSF of Patients With Neuro‐COVID‐19

CSF and serum samples from study participants were screened for IgG anti‐Spike. Most patients had detectable levels in serum (26/31, 84%), without differences in distribution between groups (Fisher's exact test, *p* = 0.6919), and CSF samples were negative, except for one patient with encephalopathy (Figure 1). However, this individual was positive for serum IgG anti‐Spike, and the CSF had 5547 RBC/mm^3^, suggesting that CSF IgG anti‐Spike derived from blood.

**Figure 1:**
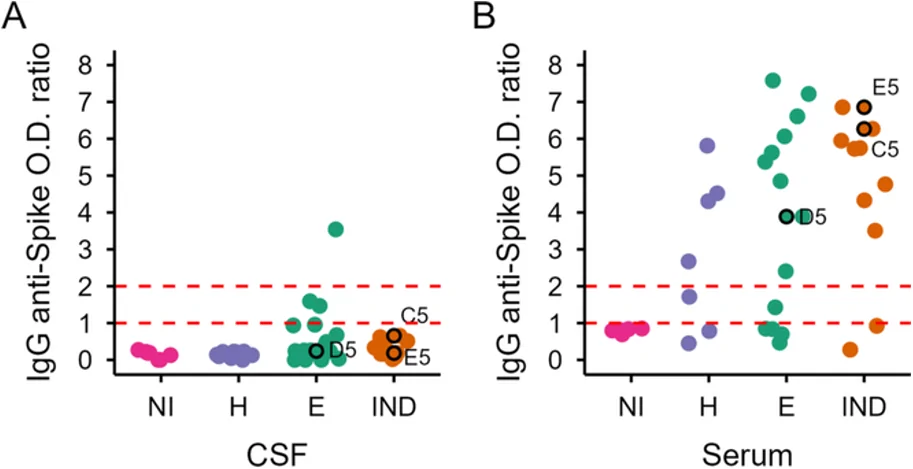
Cerebrospinal fluid (CSF) and serum screening for IgG anti‐Spike. (A) CSF samples from COVID‐19 patients with isolated refractory headache (H, *n* = 12), encephalopathy (E, *n* = 23), and inflammatory neurological diseases (IND, *n* = 12), and the control group of non‐infected individuals (NI, *n* = 7), and (B) paired serum samples available (H, *n* = 7; E, *n* = 14; IND, *n* = 10; NI, *n* = 5) were evaluated by ELISA [32]. Dashed red lines indicate optical density (OD)/cut‐off ratio values for negative (< 1.0) and positive results (> 2.0). Results between 1.0 and 2.0 OD ratios were considered indeterminate. Three patients who developed neurological symptoms more than 14 days after COVID‐19 onset are indicated: D5 (encephalopathy, 18 days), C5 (ADEM, 19 days), and E5 (acute myelitis, 29 days).

### 3.3 CSF Proteomic Changes Associated With Distinct Neuro‐COVID‐19 Manifestations

CSF obtained from patients with neuro‐COVID‐19 were investigated by proteomic analysis. Proteomic profiles presented low variability, as shown in the PCA plot (Figure 2A), independently of the neurological condition, and no clear difference was observed from NI individuals, as demonstrated by the overlap between groups. This is corroborated by the elevated number of proteins shared between groups and the low number of proteins solely detected in each group when considering all proteins identified by two unique peptides or at a threshold for sample coverage set to 33%, shown in Venn diagrams (Figure 2B). Relative expression analysis revealed a small number of differentially expressed proteins (DEPs) between neuro‐COVID‐19 groups and the control (Figure 2C−E). This included factors of the complement system (C1QA, C1QB, C1QC, C1R, C8B, and CFB), and proteins involved in the function and activation of microglia/macrophages (CD163 and LYZ), in inflammatory responses (TIMP1, B2M, CHI3L), and in the function and homeostasis of neurons (PTPRN2, SEZ6L2, NELL2, PTPRS, LY6H, CACNA2D1, NPTXR, GFRA2, and NPY). Significant differences were also observed between neuro‐COVID‐19 groups (Figure 2F).

**Figure 2:**
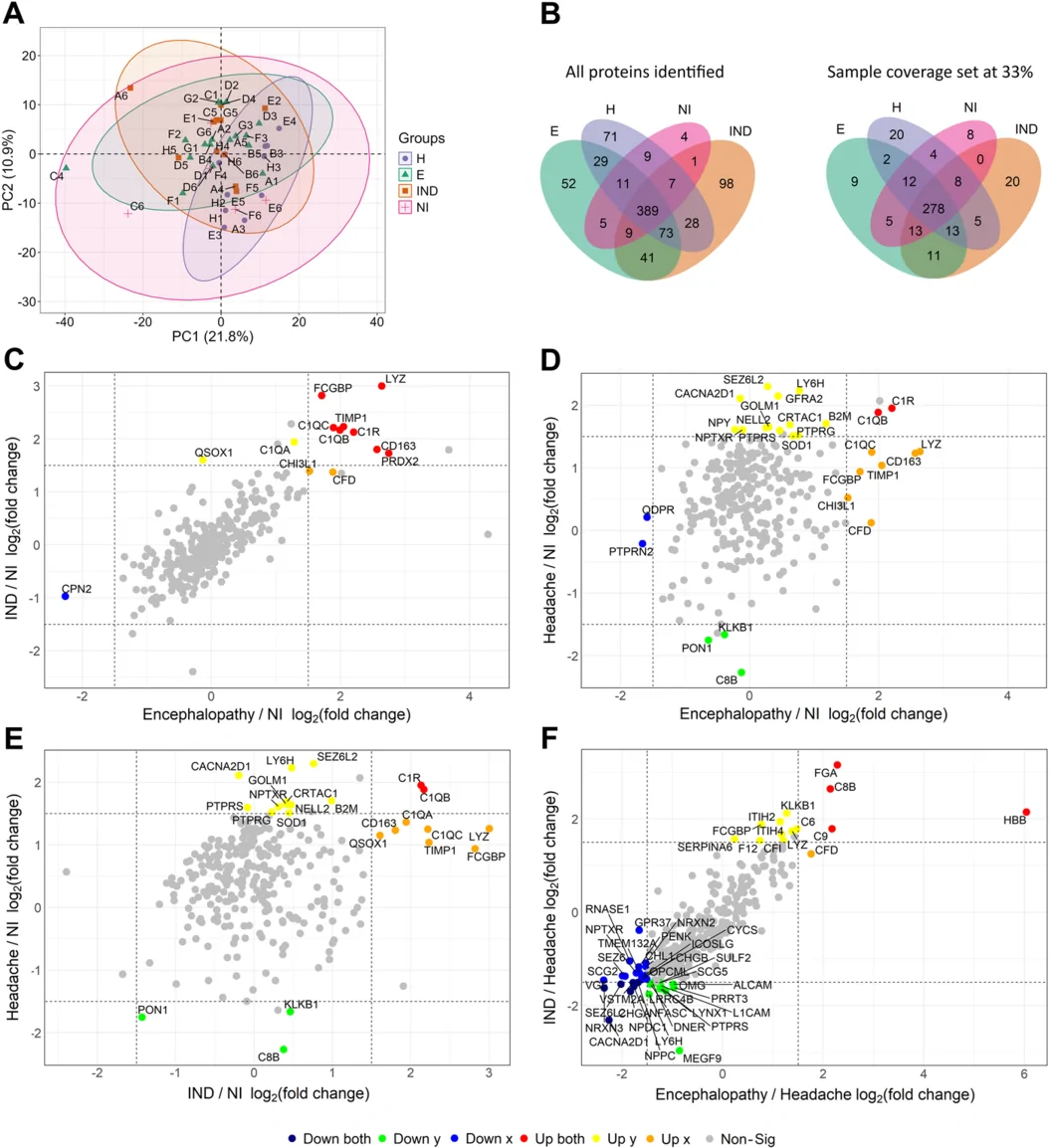
Principal component analysis (PCA) and differential expression of CSF proteins. CSF samples from patients with COVID‐19 and isolated refractory headache (H, *n* = 10), encephalopathy (E, *n* = 17), and inflammatory neurological diseases (IND, *n* = 11), and a control group of non‐infected individuals (NI, *n* = 6) were evaluated by untargeted proteomic analysis. Proteins were identified by at least two unique peptides detected in a sample. (A) A PCA plot was generated with CSF proteomic profiles obtained at a threshold for sample coverage set to 33%. Three patients who developed neurological symptoms more than 14 days after COVID‐19 onset are: D5 (encephalopathy, 18 days), C5 (ADEM, 19 days), and E5 (acute myelitis, 29 days). (B) Venn diagrams were constructed with data of all proteins identified or filtered at a threshold of 33% for sample coverage. (C–F) Differential expression analysis of CSF proteins between the study groups is shown in four‐quadrant plots using proteomic profiles obtained at a sample coverage set at 33%. Proteins downregulated in both groups are shown in dark blue, and those downregulated only in the *y*‐axis and *x*‐axis are shown in green and blue, respectively. Proteins upregulated in both groups are shown in red, and those upregulated only in the *y*‐axis and *x*‐axis are shown in yellow and orange, respectively. Proteins with nonsignificant changes in expression are shown in gray.

Functional enrichment analysis of biological processes showed that upregulated proteins in patients with encephalopathy and IND compared to NI individuals were mostly associated with complement and coagulation cascades (hsa04610), particularly the classical pathway of complement activation (GO: 0006958), and synapse pruning (GO:0098883) (Figure 3A). In contrast, processes associated with complement and coagulation cascades (hsa04610) were downregulated in patients with isolated refractory headache compared to controls (Figure 3A). The analysis of DEPs between neuro‐COVID‐19 groups showed a downregulation of factors associated with processes of adult behavior (GO:0030534) and locomotory behavior (GO:0007626), and an upregulation of those related to complement activation and coagulation cascades (hsa04610) and complement activation by alternative pathway (GO:0006957) in encephalopathy patients compared to individuals with isolated headache (Figure 3B). IND patients showed a downregulation of factors involved in synapse organization (GO:0050808) and cell−cell adhesion (GO:0098609 and hsa04514) compared to patients with isolated headache, but an upregulation of proteins related to processes of complement activation, both classical (GO:0006958) and alternative pathways (GO:0006957), of blood coagulation, including the intrinsic pathway (GO:0007597), plasminogen activation (GO:0031639), factor XII activation (GO:0002542), fibrinolysis (GO:0042730), and fibrin clot formation (GO:0072378), and of acute inflammatory response (GO:0002526) (Figure 3B). However, no significant changes were observed in the comparison between patients with IND and encephalopathy.

**Figure 3:**
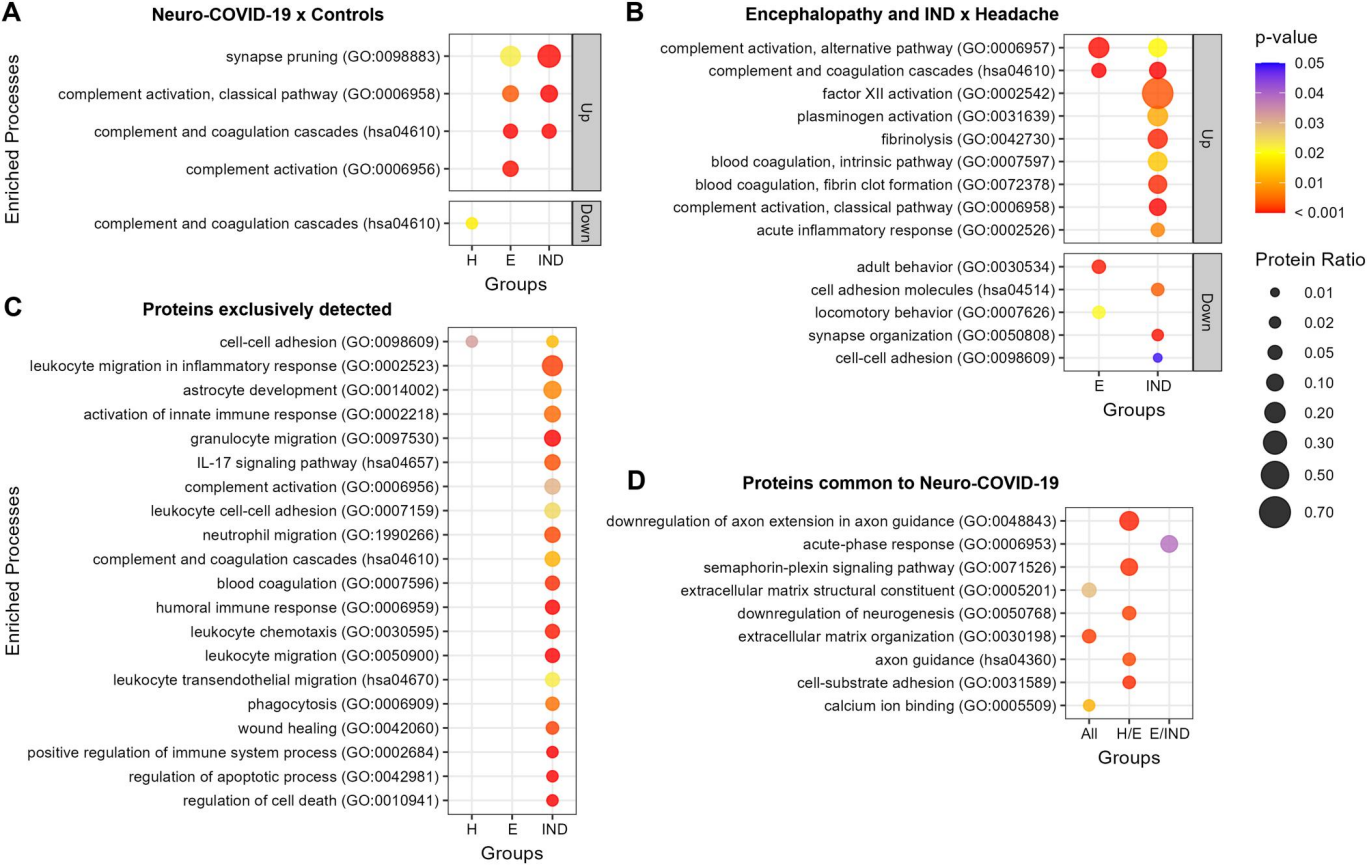
Functional enrichment analysis of proteins identified in cerebrospinal fluid (CSF) samples from patients with COVID‐19 and neurological manifestations. The enrichment of biological processes was analyzed using the STRING database. The analysis included Gene Ontology (GO) and KEGG (hsa) terms. (A) Biological processes associated with differentially expressed proteins (DEPs) in CSF according to neurological outcomes—isolated refractory headache (H), encephalopathy (E), and inflammatory neurological diseases (IND)—compared to non‐infected controls, and (B) between patients with encephalopathy and IND and patients with isolated headache. (C) Biological processes were also analyzed considering the proteins exclusively detected in each study group when including all proteins identified by two unique peptides, or (D) detected in all three neuro‐COVID19 groups, or in both groups of patients with isolated headache and encephalopathy (H/E) or with encephalopathy and IND (E/IND). Biological processes with significant enrichment (FDR test, *p* < 0.05) are shown. The protein ratio represents the proportion of proteins in the query relative to the total number associated with a given biological process.

Considering that individual proteomic profiles were diverse (Figure 2A), functional enrichment analysis of biological processes was also carried out with proteins solely detected in each study group when including all proteins identified by two unique peptides (Figure 2B) to assess grouped data associated with individual neurological manifestations in COVID‐19. The analysis of 52 proteins detected only in the CSF of patients with encephalopathy indicated no enrichment for a specific biological process, whereas the evaluation of 71 proteins detected in CSF samples from patients with isolated refractory headache showed an enrichment only for factors associated with cell‐cell adhesion (GO:0098609). In contrast, the analysis of 98 proteins detected only in the CSF of patients with IND showed an enrichment for several biological processes, with the top 20 most relevant condensed into: (1) migration of immune cells (Leukocyte migration [GO:0050900], Leukocyte chemotaxis [GO:0030595], Leukocyte migration in inflammatory response [GO:0002523], Leukocyte transendothelial migration [hsa04670], Granulocyte migration [GO:0097530], Neutrophil migration [GO:1990266]); (2) immune response (Humoral immune response [GO:0006959], Positive regulation of immune system process [GO:0002684], Activation of innate immune response [GO:0002218], IL‐17 signaling pathway [hsa04657], Complement activation [GO:0006956], Phagocytosis [GO:0006909], Leukocyte cell−cell adhesion [GO:0007159]); (3) cell death (Regulation of cell death [GO:0010941] and Regulation of apoptotic process [GO:0042981]); (4) tissue remodeling (Blood coagulation [GO:0007596], Cell−cell adhesion [GO:0098609], Wound healing [GO:0042060], and Complement and coagulation cascades [hsa04610]); and (5) CNS homeostasis (Astrocyte development [GO:0014002]) (Figure 3C).

To identify biological processes common to the neurological outcomes assessed in COVID‐19, functional enrichment analysis was performed with 73 proteins detected in all three groups of neuro‐COVID‐19 patients but not in NI controls. Extracellular matrix organization (GO:0030198) was the only biological process enriched, including factors with molecular functions defined as Extracellular matrix structural constituent (GO:0005201) and Calcium ion binding (GO:0005509) (Figure 3D). The analysis of biological processes involving proteins detected in the CSF of both IND and encephalopathy patients (41 proteins) revealed the functional enrichment only for Acute‐phase response (GO:0006953), whereas those proteins detected in both encephalopathy and isolated refractory headache (29 proteins) were associated with several biological processes, most of them related to the organization/formation of neuron projections, including Axon guidance (hsa04360), Downregulation of axon extension in axon guidance (GO:0048843), Semaphorin‐plexin signaling pathway (GO:0071526), Cell‐substrate adhesion (GO:0031589), and Downregulation of neurogenesis (GO:0050768) (Figure 3D).

### 3.4 Complement Activation Is Associated With Encephalopathy and IND in COVID‐19

Activation of the complement system in the CNS during neuro‐COVID‐19 was assessed by quantification of C3a and soluble C5b‐9 levels in CSF samples. C3a production indicates early complement activation, while C5b‐9 constitutes the membrane attack complex, reflecting the later stages of complement cascade activation. The association between complement activation and encephalopathy and IND was confirmed by higher levels of C3a and soluble C5b‐9 in the CSF of these patients compared to individuals with isolated refractory headache and uninfected controls (Figure 4).

**Figure 4:**
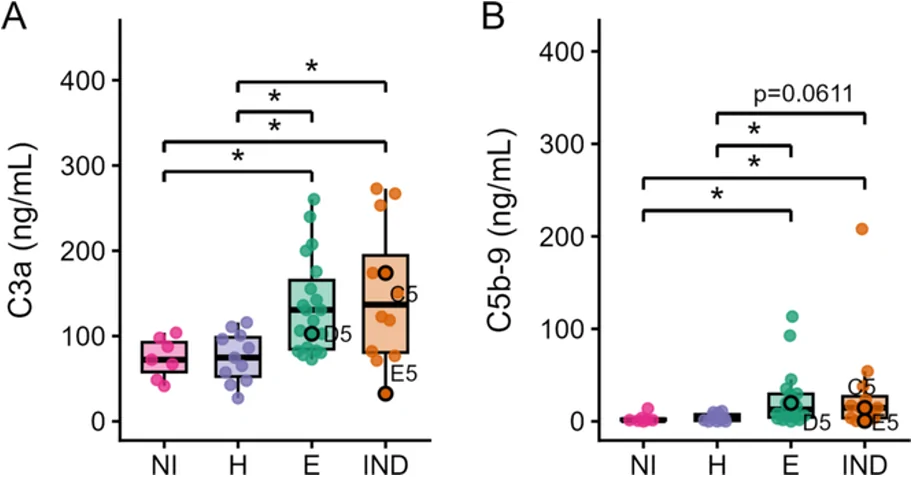
Quantification of factors derived from complement activation in the central nervous system in neuro‐COVID‐19. Cerebrospinal fluid (CSF) samples from COVID‐19 patients with isolated refractory headache (H, *n* = 12), encephalopathy (E, *n* = 23), and inflammatory neurological diseases (IND, *n* = 12), and a control group of non‐infected individuals (NI, *n* = 7) were evaluated by (A) ELISA for the quantification of C3a, and by (B) Luminex to determine soluble C5b‐9 levels. Concentration values in ng/mL are presented as median and interquartile range (IQR). Comparative analysis was performed with the Kruskal‐Wallis test and post hoc analysis using Dunn's test with Holm−Sidak method for adjusting *p* values in multiple comparisons. Results were considered significant at adjusted *p* < 0.05. *p < 0.05; **p < 0.01. Three patients who developed neurological symptoms more than 14 days after COVID‐19 onset are indicated: D5 (encephalopathy, 18 days), C5 (ADEM, 19 days), and E5 (acute myelitis, 29 days).

### 3.5 CNS Affected Regions According to CSF Changes Observed in Neuro‐COVID‐19

CSF consists of proteins that are either secreted by CNS tissue or that cross the BBB from the bloodstream. Changes in CSF composition can indicate altered brain expression, and in most COVID‐19 cases, this occurred without detectable viral CNS infection [9, 17]. Therefore, we analyzed DEPs and proteins detected in specific neuro‐COVID‐19 groups according to 13 main regions described for the human brain, as reported in the Human Protein Atlas database, to define CNS regions likely affected in neuro‐COVID‐19. This analysis found that 35 proteins were associated with at least one brain region (Figure 5A,B) and that 161 proteins were selectively expressed by at least one CNS resident cell type (Figure 5C,D).

**Figure 5:**
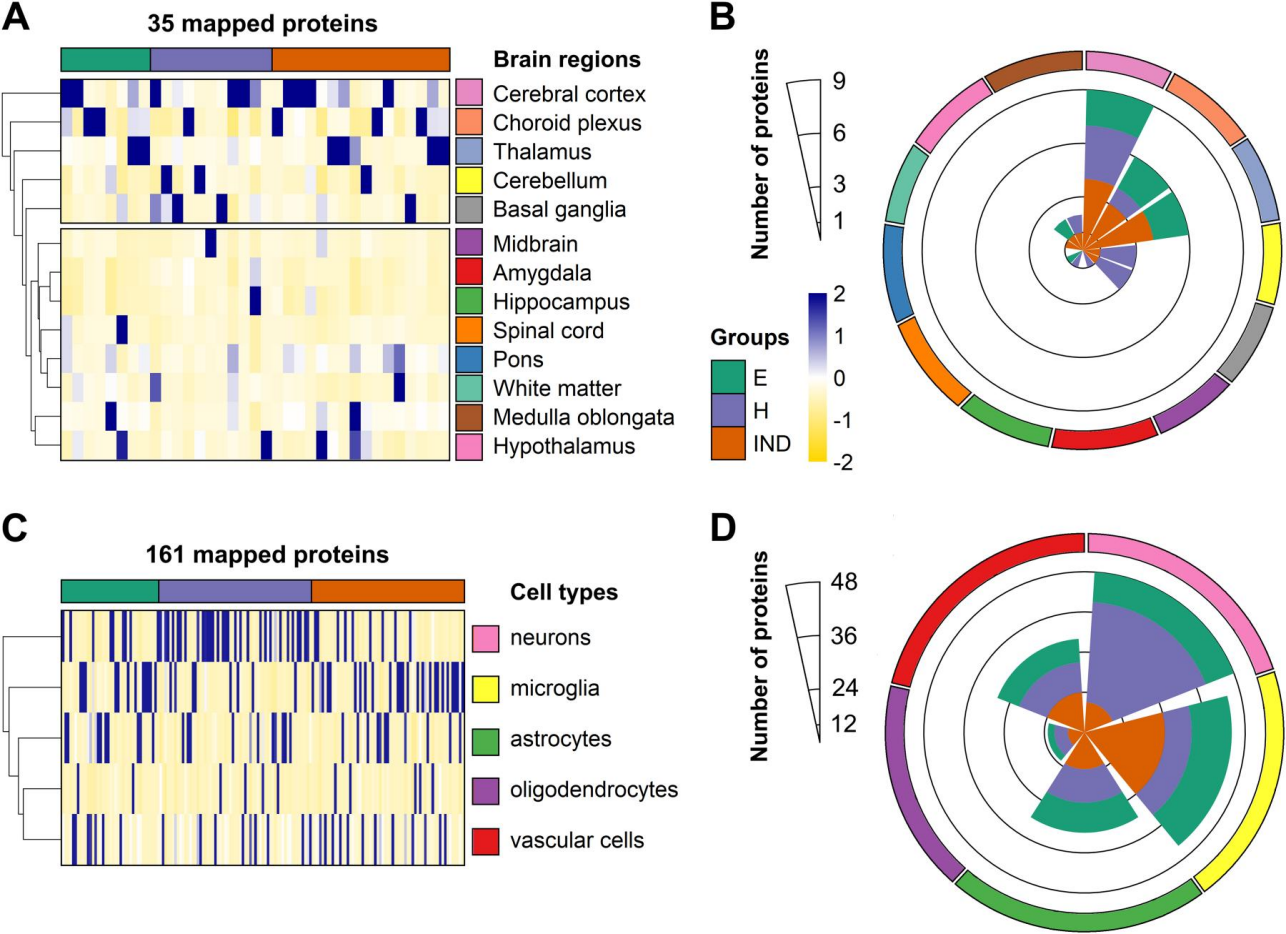
Mapping of cerebrospinal fluid (CSF) proteins identified by proteomic analysis to central nervous system (CNS) regions and resident cell types. CSF proteins differentially expressed in patients with COVID‐19 and isolated refractory headache (H), encephalopathy (E), and inflammatory neurological diseases (IND) compared to non‐infected individuals, or detected in a specific study group, were mapped to 13 CNS regions and five CNS cell types using normalized RNA expression data available at the Human Brain Atlas database (https://www.proteinatlas.org/humanproteome/brain). CSF proteins were associated with one or more brain regions and CNS cell types when the expression was fourfold or greater than the mean expression in all other brain regions and cell types. (A, C) Heatmaps show the relative expression of proteins mapped to specific brain regions and distinct CNS cell types according to neuro‐COVID‐19 groups, in which dark blue, white, and yellow indicate high, median, and low expression, respectively. (B, D) Rose plots show the number of proteins mapped to specific brain regions and CNS cell types according to neuro‐COVID‐19 groups.

CSF from patients with isolated refractory headache was enriched for proteins mapped to the cerebral cortex, cerebellum, and basal ganglia, while brain tissue‐mapped proteins in the CSF from patients with encephalopathy and IND were mainly associated with the cerebral cortex, choroid plexus, and thalamus (Figure 5A,B), indicating the involvement of cortical and subcortical structures of the brain. Proteins were also mapped to CNS resident cells, showing that the CSF from patients with isolated refractory headache was mostly enriched for proteins derived from neurons, while the CSF from patients with IND had an increased proportion of proteins mapped to microglia. In contrast, CSF from patients with encephalopathy did not show the enrichment of mapped proteins to a specific cell type (Figure 5C,D). However, definitive attribution of these proteins to their specific sources requires labeling experiments that can differentiate proteins based on their cellular or anatomical origins.

## 4 Discussion

In this study, we observed that the CSF of patients with COVID‐19 presenting with distinct neurological manifestations have heterogeneous proteomic profiles within a specific clinical condition despite the limited number of changes in CSF composition. Most changes were associated with the differential expression of factors of the complement system, markers of inflammation, function, and activation of microglia/macrophages, and the function and homeostasis of neurons. Dysregulated activation of complement was associated with IND and encephalopathy, as reflected by elevated C3a and soluble C5b‐9 levels in CSF. However, the relative contribution of its protective and pathological roles remains unclear. Enrichment of factors associated with disrupted synapse organization was observed in cases of isolated refractory headache and encephalopathy. Moreover, changes in the CSF of patients with IND were consistent with neuroinflammation, with the enrichment of factors associated with innate immune responses, cell death, tissue remodeling, migration of immune cells, and astrocyte functions, including the enrichment of microglia‐mapped proteins. These findings are in line with our previous observations and data in the literature, in which immune hyperactivation and dysregulation of coagulation pathways have been associated with brain injury in response to infection.

Neuro‐COVID‐19 patients commonly have elevated CSF levels of glial fibrillary acidic protein, neurofilament light chain protein, and total Tau protein [9, 35], consistent with astrocyte activation and neuronal injury in COVID‐19. The investigation of COVID‐19 mild and severe hospitalized patients has revealed that heterogeneous neurological symptoms are dissociated from the lung burden [36, 37]. In critically ill‐hospitalized patients with new‐onset neurological symptoms/signs, including coma, headache, acute encephalopathy, epilepsy, meningeal signs, and psychiatric disorders, the CSF and serum proteomic profiles presented low correlation, suggesting that CNS changes were likely related to intrinsic processes rather than systemic inflammation [38]. Moreover, it has been shown that systemic inflammation and CSF proteome changes related to inflammation, activation of complement and coagulation cascades, innate immunity, and hemostasis are not associated with COVID‐19 severity or neuroimaging alterations, pointing to neuroinflammation as the main driver of neurological symptoms in COVID‐19 [36]. In our cohort, however, the uneven distribution of COVID‐19 severity across neurological subgroups and the limited sample size precluded robust correlation analyses between protein levels and clinical severity scores. Serum proteomic and metabolomic changes have also been associated with dysregulated macrophage function, platelet degranulation, complement activation, and metabolic suppression [39]. The post‐mortem investigation of brain tissue also supports microglial and complement involvement in COVID‐19 [40, 41]. In another study, microglial dysfunction was linked to mitochondrial injury at sites of excessive synapse and myelin phagocytosis and loss of glutamatergic terminals [42]. Indeed, complement activation and synapse tagging in the adult CNS have been shown to be involved in synaptic elimination by microglia, as demonstrated in animal models of neuroinfectious diseases [43, 44].

Single‐cell RNA sequencing (scRNA‐seq) data from COVID‐19 patients with neurological symptoms but showing unremarkable brain MRI and CSF markers, including seizures, impaired consciousness, encephalopathy, and refractory headache, also confirmed distinct responses between the CNS and periphery. Anti‐Spike and Nucleocapsid IgG can be detected in the CSF and plasma of neuro‐COVID‐19 patients, although epitopes targeted by CSF and blood SARS‐CoV‐2 IgG may diverge. As previously investigated in our study population, only three patients had CSF‐restricted OCB, revealing intrathecal IgG synthesis, and the other three showed identical OCB in CSF and serum, indicating systemic inflammation [9]. Moreover, all CSF samples were negative for IgM anti‐Spike [28]. Therefore, since none of the patients were truly positive for CSF IgG anti‐Spike, the OCB detected was likely not associated with intrathecal anti‐SARS‐CoV‐2 humoral responses. In a mouse model that expresses human ACE2 in the brain and/or lungs, intrathecal SARS‐CoV‐2 antibodies were present only during brain infection and not elicited by pulmonary infection [45]. However, although two patients had detectable SARS‐CoV‐2 RNA in CSF [9], intrathecal anti‐Spike IgG was not detected. These findings do not support a major role for anti‐Spike IgG in neuro‐COVID‐19 mechanisms, and are not sufficient to establish viral CNS infection as a determinant of BBB alterations.

Brain regions and CNS cell types mapped to proteome changes observed in the CSF of neuro‐COVID‐19 patients were in accordance with clinical conditions. Although not identical to the brain tissue, CSF is produced by the choroid plexus and bathes the CNS. Therefore, changes in CSF composition may reflect altered brain expression. The CSF of patients with isolated refractory headache presented enrichment of proteins mainly expressed by neurons and mapped to the cerebral cortex, cerebellum, and basal ganglia. In contrast, most proteins differentially expressed or selectively detected in the CSF of patients with encephalopathy and IND were mapped to the cerebral cortex, thalamus, and choroid plexus. These findings are supported by neuroimaging studies showing structural and functional changes in the frontal, temporal, parietal, and occipital cortex, with progressive thinning of cortical gray matter and overall brain atrophy. Reduced number of neuronal cells, changes in the spatial arrangement of neurons, and an increased number of glial cells in the cerebral cortex have also been described in patients with COVID‐19, consistent with glial‐mediated neuroinflammation contributing to neuronal injury [46]. Frequent involvement of corpus callosum, cerebellum, basal ganglia, and thalami has also been reported [37, 47, 48, 49, 50, 51]. Additional findings include leptomeningeal enhancement, diffuse brain microhemorrhages, acute ischemic strokes, signs of cerebral vasculitis, and acute inflammatory demyelinating lesions [52, 53]. Cortical involvement in subacute encephalopathy has been attributed to transient dysregulation of vasomotor reactivity [54].

Inflammasome and microglial activation appear to be involved in the neuropathology of encephalopathy and IND in COVID‐19, as reflected by increased markers of glial activation, such as CD163, and elevated CSF levels of IL‐1β and IL‐18, which is consistent with NLRP3 inflammasome activation [9, 14, 28, 29, 40]. Neuropathological changes in COVID‐19 have been attributed to a gliovascular failure driven by SARS‐CoV‐2‐induced inflammation, even without evidence of direct viral invasion of the CNS [42, 55]. SARS‐CoV‐2 RNA is rarely detected in the CSF of COVID‐19 patients [17, 56]. However, viral RNA and antigens have been identified in the brain parenchyma [14]. In a post‐mortem case series, the presence of SARS‐CoV‐2 RNA in the CNS tissue did not correlate with the severity of neuropathological alterations, which included ischemic lesions, astrogliosis, microglial activation, and cytotoxic T cells infiltration in the brainstem, cerebellum, and meninges [14]. Although neuroinflammatory responses in acute COVID‐19 occur without confirmation of viral RNA in CSF, transient or tissue‐restricted neuroinfection cannot be excluded. In our study, only two CSF samples were positive in SARS‐CoV‐2 RT‐qPCR, showing high cycle threshold values, suggestive of low viral load [9].

Previous findings in this study population on dysregulated blood and/or CSF levels of TGF‐α, VEGF, and GM‐CSF, which are cytokines involved in endothelial activation, increased vascular permeability, and BBB disruption, support the role of cerebrovascular alterations in neuro‐COVID‐19 pathogenesis [28, 29]. In patients with parainfectious neurological alterations, increased choroid plexus volume has been shown as a brain structural change reflecting CNS inflammation and barrier dysfunction [57]. Moreover, infection of choroid plexus epithelial and ependymal cells at the CSF‐brain interface by SARS‐CoV‐2 has also been demonstrated [58]. The choroid plexus is a highly specialized structure located within the cerebral ventricles, consisting of a dense capillary network lined by fenestrated endothelial cells and overlaid by a monolayer of ependymal cells. Its primary functions involve CSF production and regulation of molecular and metabolic exchange between the CNS and the peripheral circulation [59, 60]. Additionally, the choroid plexus has a pivotal role in CNS immune surveillance, as macrophages and T‐cells are frequently located nearby, contributing to pathogen detection and the orchestration of inflammatory cells entry into the CNS [61]. Therefore, the choroid plexus constitutes a key component in maintaining the integrity of both the BBB and the blood‐CSF barrier. The CSF profile in neuro‐COVID‐19 patients has been characterized by markers of blood‐CSF barrier disruption, compatible with cerebrospinal endotheliopathy [22, 56]. scRNA‐seq analysis of distinct brain regions of COVID‐19 patients also identified an increased proportion of stromal cells, monocytes, and macrophages in the choroid plexus [62]. Our previous findings showed a marked CSF cytokine storm in patients with encephalopathy, in which biochemical, cellular, and imaging signs of acute brain inflammation were absent, suggesting a temporal sequence in which neurological manifestations may arise from CNS responses to systemic inflammation, rather than from primary CNS involvement, which in turn was associated with cases of IND [9, 28, 29]. Thus, increased levels of CSF proteins mapped to the choroid plexus in COVID‐19 patients may reflect persistent barrier dysfunction and be associated with altered immune trafficking in IND cases and the exposure of the CNS to circulating inflammatory mediators in encephalopathy. In contrast, isolated refractory headache was associated with intracranial hypertension [6]. However, the mechanisms underlying impaired CSF volume regulation remain unclear.

This study has limitations, including a small sample size, data variability, limited CSF volume to perform validation assays for all proteomic findings, a lack of consecutive CSF and serum samples, and the lack of long‐term follow‐up of neuro‐COVID‐19 patients to determine whether they resolved or developed post‐COVID conditions, which could aid in identifying early disease biomarkers. Patient groups could not be adjusted for age, sex, and underlying clinical conditions, which may represent potential confounders. However, our findings are supported by previous studies and align with existing evidence on neuroinflammation, microglial responses, BBB dysfunction, complement activation, and neuronal injury in COVID‐19. Strengths of this study include a comprehensive investigation of neuroinflammation in a well‐characterized cohort with diverse neurological outcomes, supported by extensive prior data on biomarkers of inflammation and neuronal damage, and the inclusion of uninfected controls, allowing the identification of biological processes associated with neurological manifestations in acute neuro‐COVID‐19.

## 5 Conclusions

In conclusion, systemic inflammation in COVID‐19, in addition to activation of the complement cascade in the CNS, was associated with altered markers of endothelial activation and BBB disturbances, which may result in microglial and astrocyte activation in cases of acute neuroinflammatory syndromes and encephalopathy. Findings from this study, and previous experimental and clinical evidence, are consistent with a pathophysiological model in which neuroinflammatory processes promote a CNS cytokine storm and/or immune cell trafficking, which may further contribute to BBB disruption and neuronal injury in the context of distinct acute neurological manifestations, particularly encephalopathy and IND.

## Author Contributions

Nicole Lardini Freitas performed sample preparation for proteomic analysis, conducted ELISA, data analysis, and drafted the manuscript. Yago Côrtes Pinheiro Gomes performed proteomic data analysis. Fábio César Sousa Nogueira performed liquid chromatography‐tandem mass spectrometry analysis. Juliana Echevarria‐Lima performed IgG anti‐SARS‐CoV‐2 ELISA. João Victor Carvalho Deus carried out functional network analysis. Carlos Otávio Brandão performed CSF and paired serum laboratory analysis. Cristiane Nascimento Soares and Marcus Tulius Teixeira Silva acquired clinical data and conducted neurological assessments. Nicole Lardini Freitas, Yago Côrtes Pinheiro Gomes, João Victor Carvalho Deus, and Otávio Melo Espíndola assembled the figures. Otávio Melo Espíndola contributed to the study concept and design, performed Luminex assays, data analysis, supervised all phases of the project, and drafted the manuscript. All authors contributed to the interpretation of the results, read and approved the final manuscript.

## Ethics Statement

The study protocol was approved by the Brazilian National Committee of Ethics in Research on April 22, 2020 (Protocol number 30611720.6.0000.5262), and written informed consent was obtained from all participants. All methods were performed in accordance with the relevant guidelines and regulations. Access to the genetic heritage of SARS‐CoV‐2 under investigation is registered in the Brazilian National System for the Management of Genetic Heritage and Associated Traditional Knowledge (SisGen A53F54D).

## Conflicts of Interest

The authors declare no conflicts of interest.

## Supporting information


Supporting File

## Data Availability

The data that support the findings of this study are openly available in the Proteomics Identifications Database (PRIDE) repository at https://www.ebi.ac.uk/pride, reference number PXD067176.
