# Supplementary material for: Acute Neurological Manifestations in COVID‐19 Are Associated With Complement Activation and Glia‐Promoted Neuroinflammation
Source: J Med Virol. 2026 Sep 28;98(10):e71165. doi: 10.1002/jmv.71165 (PMC13618267; doi:10.1002/jmv.71165)
Supplement: Supplementary file 1 [file JMV-98-e71165-s001.pdf]

Characterization of the CSF proteomics workflow

To evaluate CSF changes that may contribute to and/or be a consequence of the CNS pathology observed in patients with COVID-19, we carried out untargeted proteomic analysis. Spectral libraries identified 1154 proteins of which 827 were identified with at least two unique peptides in a CSF sample (Table S1). Data from four participants (one individual with refractory headache, one with encephalopathy and two with IND) were excluded from further analysis due to the small number of proteins identified (less than 30 proteins) (Fig. S1A). CSF samples from non-infected individuals (n=6), patients with isolated refractory headache (n=10), encephalopathy (n=17), and IND (n=11) had a total of 435, 617, 609, and 646 proteins identified by two unique peptides, respectively (Table S1).

Table S1. Number of proteins identified in the CSF of COVID-19 patients by proteomics.

Coverage of samples	Total number of proteins identified per group				Shared/total proteins (%)
	H	E	IND	NI	
At least 1	617	609	646	435	389/827 (47%)
10%	617	488	464	435	369/700 (52%)
25%	394	358	389	328	298/457 (65%)
33%	342	343	348	328	278/408 (68%)
50%	305	283	297	290	241/353 (68%)
75%	225	211	219	192	170/259 (65%)
90%	205	149	187	137	118/221 (53%)
All	173	115	153	137	96/201 (47%)

Notes: H, headache; E, encephalopathy; IND, inflammatory neurological diseases; NI, non-infected control group.

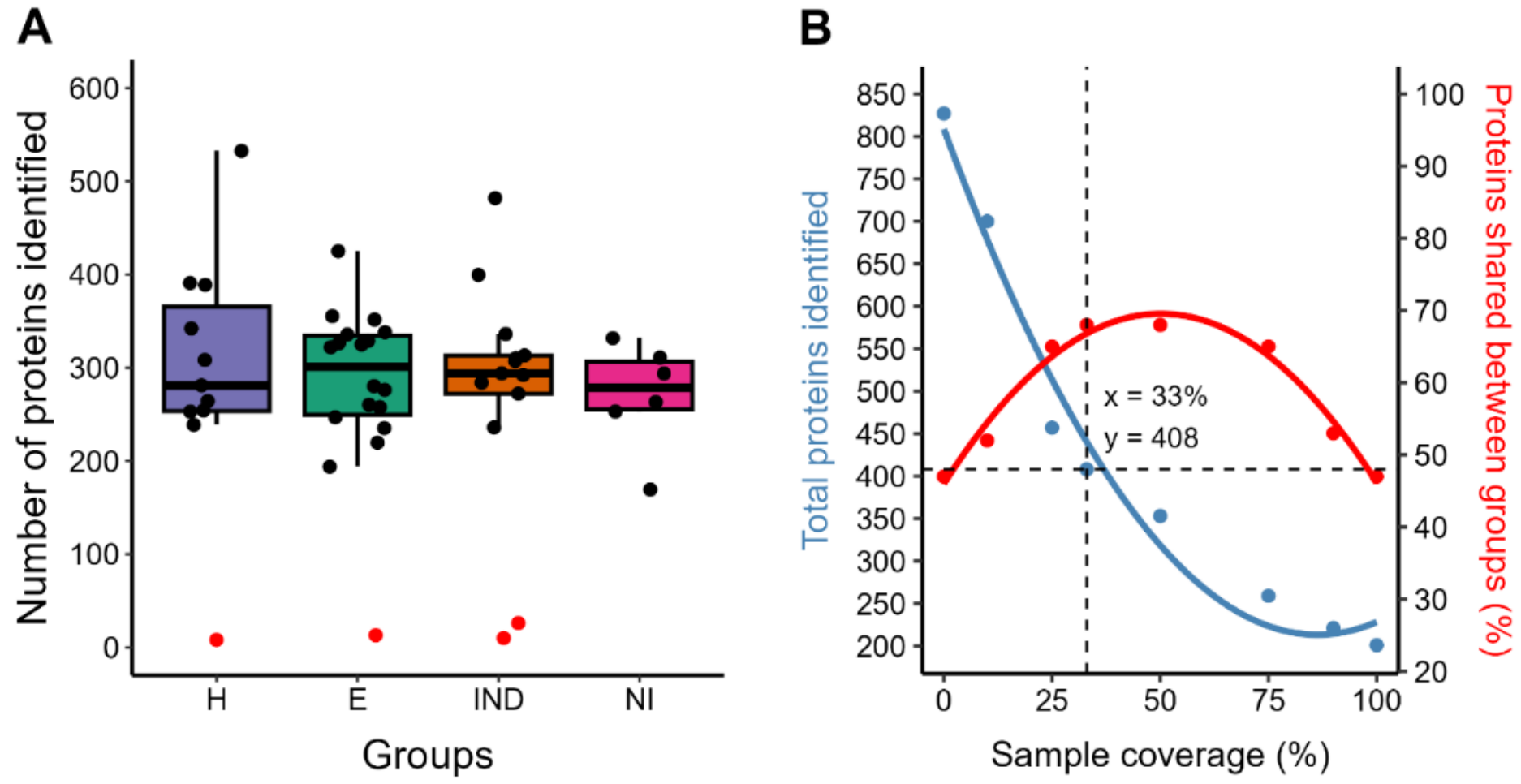

Fig S1. Cerebrospinal fluid (CSF) proteins identified by proteomic analysis. (A) CSF samples from COVID-19 patients with isolated refractory headache (H, n=11), encephalopathy (E, n=18), and inflammatory neurological diseases (IND) (n=13), and a control group of non-infected individuals (NI, n=6) were evaluated by untargeted proteomics analysis. Proteins were identified with at least two unique peptides in a sample. Points in red represent samples with less than 30 proteins identified and therefore excluded from further analysis. **(B)** Data completeness was evaluated by the total number of proteins identified according to sample coverage (blue line), which is the minimum percentage of samples that share a maximum number of proteins, and by the percentage of proteins shared between groups (red line).

Proteomic data was evaluated for data completeness. Thus, considering proteins identified in at least one sample, 47% of them were shared between all study groups (Table S1). However, by progressively increasing the minimum percentage of samples that share a maximum number of proteins, there was a decline in the number of proteins detected, but an increasing homogeneity of proteins identified among groups (Fig. S1B). This was shown by the proportion of proteins shared by all study groups in the total number of proteins identified, peaking at 68% when the threshold for sample coverage was set to 33% (Table S1 and Fig. S1B). At this threshold, the total number of proteins identified (n = 408) was reduced by half.

Therefore, for further analysis, including the identification of differentially expressed proteins between groups, clustering analysis, functional protein network, and enrichment of biological processes, it was chosen to work with proteins identified by two unique peptides in one-third (33%) of the samples.
